# Exploring Dual-Targeted Therapy in the Management of Moderate to Severe Inflammatory Bowel Disease: A Retrospective Study

**DOI:** 10.1093/crocol/otae057

**Published:** 2024-10-23

**Authors:** Sonya Bhaskar, Zachary Makovich, Rahul Mhaskar, Emily Coughlin, Jennifer Seminerio-Diehl

**Affiliations:** Division of Digestive Diseases and Nutrition, Morsani College of Medicine, University of South Florida, Tampa, FL, USA; Division of Digestive Diseases and Nutrition, Morsani College of Medicine, University of South Florida, Tampa, FL, USA; Division of Digestive Diseases and Nutrition, Morsani College of Medicine, University of South Florida, Tampa, FL, USA; Division of Digestive Diseases and Nutrition, Morsani College of Medicine, University of South Florida, Tampa, FL, USA; Division of Digestive Diseases and Nutrition, Morsani College of Medicine, University of South Florida, Tampa, FL, USA

**Keywords:** inflammatory bowel disease, Crohn’s, ulcerative colitis, dual-targeted therapy, biologics, small molecules

## Abstract

**Background:**

Inflammatory bowel disease (IBD), including ulcerative colitis (UC) and Crohn’s disease (CD), often results in significant morbidity among patients with moderate to severe forms. While biologics and small molecules are effective in inducing remission, many patients experience refractory disease or extraintestinal manifestations. This study assesses the safety and efficacy of dual-targeted therapy in IBD patients treated at the Inflammatory Bowel Disease Center.

**Methods:**

This retrospective cohort study examined 79 patients with UC or CD who received dual-targeted therapy at the University from October 2018 to August 2023. Data collected included demographics, disease characteristics, previous treatments, and clinical outcomes. Primary outcomes were endoscopic, radiographic, and patient-reported clinical improvements, with secondary outcomes focusing on safety profiles.

**Results:**

Among the 79 patients (42 UC, 37 CD), 97 dual-targeted therapy cases were analyzed, primarily involving a biologic combined with a JAK inhibitor (90.7%). The median therapy duration was 39.1 weeks. Endoscopic improvement occurred in 69% of matched samples, with significant differences between pre- and postdual-targeted therapy Mayo scores for UC (*P* = .002) and Simple Endoscopic Score for CD (SES-CD) scores for CD (*P* = .018). The median pre- and postdual-targeted therapy Mayo scores across matched samples were 3 (range 1-3) and 1 (range 0-3), respectively, and for SES-CD scores were 12 (range 0-36) and 4 (range 0-20), respectively. Clinical improvement was reported by 73.2% of patients, with notable reductions in ESR (median 19 [range 2-124] mm/h to 9 [range 0-116] mm/h, *P* = .006), CRP (median 8.0 [range 0.2-78.5] mg/L to 3.0 [range 0.2-68.2] mg/L, *P* < .001), and albumin levels (4.0 [range 2.2-4.9] mg/dL to 4.2 [range 3.4-5.2], *P* < .001). Non-obesity was associated with both more endoscopic improvement (*P* = .002) and clinical improvement (*P* = .007). Adverse events occurred in 37 cases, predominantly upper respiratory tract infections and dermatologic issues, with no thromboembolic events reported.

**Conclusions:**

Dual-targeted therapy demonstrated efficacy in improving clinical and endoscopic outcomes in patients with severe, refractory IBD and exhibited an acceptable safety profile. Despite the promising results, further research is needed to confirm these findings and determine optimal therapy combinations.

## Introduction

Inflammatory bowel disease (IBD) refers to 2 chronic conditions, ulcerative colitis (UC) and Crohn’s disease (CD), which are disease states characterized by relapsing and remitting inflammation of the gastrointestinal tract.^1^ For patients with moderate to severe IBD, patients may experience high symptom burden and increased morbidity.^1^

IBD exists on a spectrum, and the treatment of moderate to severe disease involves the use of advanced therapies such as biologics or small molecules to maintain remission. Guidelines on the treatment of IBD endeavor to induce and maintain endoscopic remission, achieve steroid-free remission, and decrease the number of hospitalizations and surgeries.^2^ The recent STRIDE II guidelines focus on a treat-to-target model to obtain a clinical response and remission, the normalization of inflammatory markers (C-reactive protein [CRP] and fecal calprotectin), and endoscopic healing.^3^

While these targets can be achieved in some, other patients are refractory or nonresponsive to treatments and/or suffer a secondary loss of response over time. About 30% of patients are considered primary nonresponders, and up to 40% may lose response to therapy as secondary nonresponders.^4–6^ Patients who have had primary or secondary treatment failure of antitumor necrosis factor (TNF) therapy only have a 30% and 45% rate of response with a second anti-TNF therapy, respectively.^7^ Patients who have already received anti-TNF therapy may have a lower rate of effectiveness with vedolizumab and ozanimod.^8^ No other biologic therapies have demonstrated the rates of clinical and endoscopic remissions that are seen with TNF antagonists.^9^ Furthermore, patients who have failed multiple treatments are often excluded from clinical trials, making options limited.^9^ Other patients suffer extraintestinal manifestations (eg, arthralgias, uveitis, erythema nodosum) not controlled by the treatment that is acting favorably on their luminal disease.^10^

Thus, combining therapies with varying mechanisms of action for severe disease is appealing.^9^ The premise is that targeting different pathogenic pathways with 2 different drugs may provide an additive or synergistic benefit and overcome the mechanistic failure that can occur in patients with severe, refractory disease.^11,12^

Combining different IBD therapies has been done before with the use of biologics such as TNF antagonists and immunomodulators. However, this is associated with an increased risk of lymphoma and infection.^9^ Biologics have a better safety profile than most immunosuppressives, and some, like vedolizumab, are gut-specific.^13,14^ To this date, there are 3 randomized control trials on the efficacy and safety profile of dual-targeted therapy with the use of biologics. The first study looked at the safety and efficacy of natalizumab and infliximab in patients with active CD despite ongoing infliximab treatment.^15,16^ While the study showed clinical efficacy, it was not statistically significant, and natalizumab is not regularly used in the management of IBD given the concerns of progressive multifocal leukoencephalopathy.^15,16^ The EXPLORER trial looked at vedolizumab, adalimumab, and methotrexate therapy in biologic-naive patients with high-risk CD and found endoscopic remission and response with no increased safety signal.^16,17^ Most recently, the VEGA trial combined guselkumab and golimumab compared to guselkumab or golimumab monotherapy in patients with moderately to severely active UC found that combination therapy with guselkumab and golimumab might be more effective for UC.^16,18^ There are no randomized controlled trials that have looked at the use of dual-targeted therapy with biological and small molecule therapy. However, data regarding small molecules from rheumatological studies and post hoc analysis of IBD patients report an increased risk of herpes zoster and thromboembolic events in the induction phase.^7^

The Inflammatory Bowel Disease Center is a tertiary referral system that often treats patients with severe cases of IBD that have been refractory to multiple treatment options. We have several patients who have been initiated on different combinations of dual-targeted therapy. This is a relatively new approach, and there is limited literature on the experience, safety, efficacy, and challenges of this treatment model. We aim to share our experiences with the safety and efficacy of this treatment strategy.

## Methods

### Study Design and Population

This was a medical-record retrospective cohort study of established patients diagnosed with CD or UC who were treated with dual-targeted therapy and were a part of the IBD registry at the Inflammatory Bowel Disease Center from October 2018 to August 2023. Dual-targeted therapy is a combination of 2 biological therapies or a biological and a small molecule therapy. The patients were initiated for the following indications: (1) partial disease response to biological or small molecule (based on imaging, endoscopic evaluation), (2) partial clinical response to biologic or small molecule, or (3) extraintestinal manifestations.

### Data Collection

Patient characteristic information was collected, including sex, age, body mass index (BMI), health insurance, and smoking status. The clinical data included the presence of UC versus CD, age at diagnosis, history of prior surgeries, prior biologic, small molecule, immunomodulator therapy, prior mesalamine, and steroid use.

Pre- and postdual-targeted therapy endoscopic scores, imaging, hemoglobin, albumin, and inflammatory markers ESR (erythrocyte sedimentation rate), CRP, and fecal calprotectin were collected. Postdual-targeted therapy follow-up times, steroid use, immunomodulator use, and surgeries were noted. An extensive chart review was performed to record reasons for initiating and discontinuing dual-targeted therapy, reported adverse effects, and patient-reported improvement in gastrointestinal and extraintestinal symptoms.

One primary outcome was endoscopic improvement, defined as improvement in endoscopic scores as per the Simple Endoscopic Score for CD (SES-CD) Rutgeerts score and Mayo score. Patient-reported clinical improvements, as well as radiographic change, were the other primary outcomes. The primary outcomes of endoscopic change, radiographic change, and change in gastrointestinal symptoms judged response to therapy. These primary outcomes were graded as worsened, stable, or improved following combination therapy initiation. The endoscopic change was based on a patient’s Mayo score for UC and SES-CD or Rutgeert’s score for CD, respectively, with increasing scores assigned a “worsened” outcome and decreasing scores assigned an “improved” outcome. Unchanged Mayo or SES-CD scores were graded as “stable.” The radiographic change was based on the presence/degree of intestinal inflammation, abscesses, and fistulas when comparing pre- and postdual-targeted therapy status. Clinical improvement in gastrointestinal symptoms was based on a 2-author review of patient records, with an assessment of patient subjective responses regarding pain, bleeding, bowel movement frequency, and patient-reported typical flare symptoms. Changes in inflammatory markers (ESR, CRP, calprotectin) were also analyzed. The secondary outcome was safety defined by any side effects (any documented side effects attributed to dual-targeted therapy).

Categorical variables were compared using Pearson’s chi-square test and Fisher’s Exact test. The Mann–Whitney U test compared the distribution differences in continuous variables across the 2 treatment groups. Lab values and endoscopic scores were compared from pre- to postdual-targeted therapy with matched samples using the Wilcoxon signed-rank test. Analysis was completed using SPSS Version 29.

Data are not publicly available due to patient privacy concerns.

## Results

### Patient Characteristics

A single gastroenterologist identified 79 patients on dual-targeted therapy from the IBD registry at the University’s IBD center from October 2018 to August 2023. The analysis included 79 patients, 42 (52.6%) males and 37 (47.4%) females. From this sample, 37 (47.4%) had CD, and 42 (52.6%) had UC. The baseline median SES-CD for all CD patients was 13 (range 0-36), and baseline median Mayo score for all UC patients was 3 (range 0-3). Before dual-targeted therapy, 29 (37.6%) patients had at least one IBD-related surgery and 63 (79.7%) patients had at least one IBD-related hospitalization. The median patient age was 39 (range 20-79). Median patient BMI was 26.6 kg/m^2^ (range 17.6-57.8 kg/m^2^). Baseline patient characteristics, including a distribution of Montreal classification across all patients, are shown in Table 1.

**Table 1.** Baseline patient characteristics.

Characteristic	Value^a^			Montreal classification^b^	
Male	*n* = 42	A1 (L3 + L4) B3p	*n* = 2	A2 L3 B2p	*n* = 1
Female	*n* = 37	A1 L2 B1	*n* = 1	A2 L3 B3	*n* = 2
Median age (years)	39 (20-79)	A1 L2 B3p	*n* = 1	A2 L3 B3p	*n* = 6
Median duration of disease (years)	9 (0-45)	A1 L3 B1	*n* = 1	A3 L2 B1	*n* = 1
Median BMI (kg/m^2^)	26.6 (17.6-57.8)	A1 L3 B2	*n* = 1	A3 L3 B1	*n* = 1
Current smoker	*n* = 6	A1 L3 B2p	*n* = 1	A3 L3 B2	*n* = 1
Former smoker	*n* = 14	A1 L3 B3	*n* = 1	E1 S2	*n* = 2
Prior IBD-related hospitalizations	*n* = 63	A1 L3 B3p	*n* = 4	E2 S1	*n* = 2
Prior IBD-related surgeries	*n* = 29	A2 (L3 + L4) B3p	*n* = 1	E2 S2	*n* = 6
Patients with Crohn’s	*n* = 37	A2 L2 B1	*n* = 3	E2 S3	*n* = 2
Median (range) baseline SES-CD^c^	13 (0-36)	A2 L2 B2p	*n* = 1	E3 S1	*n* = 1
Crohn’s with fistula	*n* = 24	A2 L2 B3p	*n* = 6	E3 S2	*n* = 23
Crohn’s with stricture	*n* = 13	A2 L3 B1	*n* = 1	E3 S3	*n* = 6
Crohn’s with perianal involvement	*n* = 22	A2 L3 B2	*n* = 1		
Patients with ulcerative colitis	*n* = 45				
Median (range) baseline Mayo score^c^	3 (0-3)				

Abbreviations: BMI, body mass index; IBD, inflammatory bowel disease; SES-CD, Simple Endoscopic Score for Crohn’s disease; UC, ulcerative colitis.

^a^Values listed as frequencies (*n* = number of patients), or median (range), or average (standard deviation).

^b^Montreal Score: For Crohn’s, Age (A) of onset is denoted by A1 ≤ 16 years, A2 for 17-40 years, and A3 ≥ 40 years of age. Location (L) is categorized as L1 for ileal involvement, L2 for colonic involvement, L3 for ileocolonic involvement, and L4 upper gastrointestinal involvement. Behavior (B) is classified as B1 for nonstricturing, nonpenetrating disease, B2 for stricturing disease, and B3 for penetrating disease, with “p” as a modifier for perianal involvement. For UC, the extent of disease (E) is denoted by E1 for ulcerative proctitis, E2 for left-sided disease, and E3 for extensive disease (extending beyond the splenic flexure). Severity (S) is categorized as S0 for clinical remission, S1 for mild disease (≤4 bloody stools daily, lack of fever, pulse < 90 bpm, hemoglobin > 105 g/L, ESR < 30 mm/h) S2 for moderate disease (>4-5 stools daily but with minimal signs of systemic toxicity), and S3 for severe disease (≥ 6 bloody stools daily, pulse > 90 bpm, temperatures > 37.5 °C, hemoglobin < 105 g/L, ESR > 30 mm/h). For all frequencies *n* = number of patients.

^c^Note these values are across all patients, compared to the values in Table 4 which reflect cases with available matched sample data.

Of the 79 patients in this study, there were 97 cases of dual-targeted therapy (ie, some patients went through multiple combination therapies). The primary reason for dual-targeted therapy initiation was to achieve clinical and endoscopic improvement. However, 9 cases involved starting dual-targeted therapy mainly for extraintestinal manifestations. Of the 97 combination therapy cases, 9 (9.3%) entailed combination biologics, while the remaining 88 (90.7%) entailed a combination of a biologic plus a JAK inhibitor. The types of combination therapies used are listed in Table 2. Before dual-targeted therapy, all but one patient had been on at least one biologic; the one patient without exposure to a biologic was on tofacitinib before combination therapy. Otherwise, the average number of biologics per patient before dual-targeted therapy was 2.88. Of note, 74/79 (93.6%) patients had received TNF-alpha inhibitor therapy before dual-targeted therapy. Of these, 49/79 (62.0%) had received infliximab before dual-targeted therapy. Other precombination medication history included azathioprine or 6-mercaptopurine in 42 patients (53.2%), 5-aminosalicylic-acid derivatives in 59 patients (75.6%), and methotrexate in 20 cases (25.3%). Precombination therapy medications are listed in Table 3.

**Table 2.** List of dual-targeted therapies used.

	Combination therapy	Number of cases
Combination class	Biologic + JAK inhibitor	88
	Dual Biologic	9
Specific combination	Vedolizumab + Tofacitinib	26
	Ustekinumab + Upadacitinib	23
	Ustekinumab + Tofacitinib	21
	Vedolizumab + Upadacitinib	13
	Ustekinumab + Vedolizumab	3
	Risankizumab + Tofacitinib	3
	Ustekinumab + Infliximab	2
	Vedolizumab + Risankizumab	2
	Infliximab + Tofacitinib	1
	Ustekinumab + Certolizumab	1
	Certolizumab + Upadacitinib	1
	Vedolizumab + Certolizumab	1

**Table 3.** Prior therapies used before dual-targeted therapy.

Therapy type	Number of uses prior to combination therapy^a^
5-ASA	59
6-mercaptopurine or azathioprine	42
Methotrexate	20
Tofacitinib	15
Upadacitinib	5
Infliximab	49
Adalimumab	56
Certolizumab	8
Golimumab	4
Vedolizumab	51
Natalizumab	1
Ustekinumab	37
Risankizumab	2

^a^Each instance of prior therapy was only counted once per patient and not counted multiple times in cases where patients were on multiple combination therapies.

### Primary Outcomes: Endoscopic, Radiographic, and Patient-Reported Changes

**Figure 1.**
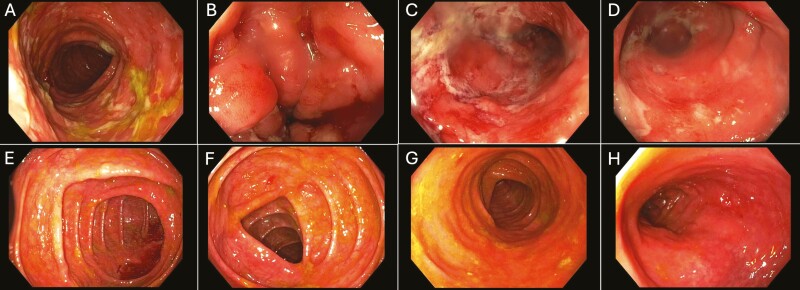
The figure represents a 21-year-old male with Crohn’s colitis with uncontrolled symptoms despite 8 months of ustekinumab therapy. Endoscopy images show predual-targeted therapy findings in the ascending colon (A), transverse colon (B), descending colon (C), and rectum (D); the composite Simple Endoscopic Score for Crohn’s disease (SES-CD) was 36. Upadacitinib was subsequently added, and the repeat endoscopy performed 7 months later demonstrated improvement in all of the respective regions of the colon (E-H), corresponding to a composite SES-CD of 4. No steroids or immunomodulators were used in the interim period.

The median duration of dual-targeted therapy was 39.1 weeks (range 4.3-272.4 weeks). The median time to a case’s last endoscopy after therapy initiation was 9 months. Of the 97 dual-targeted therapy cases, there were 42 cases with completed endoscopies and available pre- and post dual-targeted therapy Mayo scores (for UC cases) or SES-CD scores (for CD cases). There was a statistically significant difference between the pre- and post dual-targeted therapy endoscopic findings for both the Mayo scores (*P* = .002) and the SES-CD scores (*P* = .018). The median pre- and post dual-targeted therapy Mayo scores from these cases were 3 (range 1-3, interquartile range [IQR] 1.5) and 1 (range 0-3, IQR 1), respectively, and for SES-CD scores were 12 (range 0-36, IQR 13) and 4 (range 0-20, IQR 9), respectively. The breakdown of postdual-targeted therapy outcomes was as follows: 4/42 (9.5%) worsened, 9/42 (21.4%) were stable, and 29/42 (69.0%) improved. Endoscopic outcomes are summarized in Table 4. Endoscopic change in a patient with CD is illustrated in Figure 1. There was no significant difference in endoscopic improvements between CD and UC cases (*P* = 1.000). Using a BMI of 30 kg/m^2^ as a cutoff with the obesity threshold, dual-targeted therapy cases with nonobese patients were associated with significantly more endoscopic improvement than obese patients (*P* = .002).

**Table 4.** Endoscopic and clinical responses based on type of dual-targeted therapy.

			Biologic + JAK inhibitor			Dual biologics	
	Number of cases with available data	Improved	Stable	Worse	Improved	Stable	Worse
**Endoscopic response** ^a^	42						
Ulcerative colitis cases	29	19	8	1	1	0	0
Crohn’s cases	13	8	1	2	1	0	1
Clinical response^b^	82						
Ulcerative colitis cases	50	36	4	6	2	1	1
Crohn’s cases	32	19	4	5	3	1	0

Abbreviations: SES-CD, Simple Endoscopic Score for Crohn’s disease; IQR, interquartile range; UC, ulcerative colitis

^a^Endoscopic change is based on the change in Mayo score for UC cases and SES-CD score for Crohn’s disease cases. There was a statistically significant difference between pre- and post-dual-targeted therapy for Mayo scores (*P* = .002) and SES-CD scores (*P* = .018) using the Wilcoxon signed-rank test. The median pre- and post-dual-targeted therapy Mayo scores from these cases were 3 (range 1-3, interquartile range [IQR] 1.5, pretherapy) and 1 (range 0-3, IQR 1, posttherapy), and for SES-CD scores were 12 (range 0-36, IQR 13, pretherapy) and 4 (range 0-20, IQR 9, posttherapy).

^b^Clinical change is based on the subjective patient response at the follow-up visit(s) after starting dual-targeted therapy. This is based on a 2-author review of pre- and post-dual-targeted therapy patient-reported bowel movement frequency, hematochezia, and pain.

Only 16 cases had pre- and postdual-targeted therapy imaging available for review studies, including a combination of CT and MRI. Imaging changes were worse in 6 (37.6%), stable in 5 (31.2%), and improved in 5 (31.2%) of these cases.

Eighty-two cases had in-office follow-up after combination therapy to measure clinical response to dual-targeted therapy. For these patient-reported outcomes, 60/82 (73.2%) cases had reported improvement, 10/82 (12.2%) cases had reported stability, and 12/82 (14.6%) cases had reported worsening. Of these 82 cases, 74 were in the biologic plus JAK inhibitor group with the following breakdown: 55/74 (74.3%) cases had improvement, 8/74 (10.8%) cases were stable, and 11/74 (14.28%) cases had worsening. For the other 8 cases in the dual biologic group, 5/8 (62.5%) had improvement, 2/8 (25%) were stable, and 1/8 (12.5%) had worsening. The patient-reported clinical improvement did not significantly differ between those with CD versus UC (*P* = .663). Clinical response is also summarized in Table 4. Similar to endoscopic change, non-obesity was associated with more clinical improvement than cases with obese patients (*P* = .007). Patients reported that extraintestinal manifestation (joint, skin, ocular) outcomes were mostly improved or stable (Table 5).

**Table 5.** Extraintestinal manifestation patient-reported outcomes after dual-targeted therapy.

		Postcombination outcome (# cases)	
Type of extraintestinal manifestation	Improved	Stable	Worsened
Joint	8	15	5
Skin	4	1	1
Ocular	3	2	0

For the 16 patients who underwent multiple combination therapies, 6 switched combinations due to a lack of clinical response, and 5/6 of those patients improved in clinical response after the second combination trial. The remainder of the patients who underwent multiple combination therapies did so due to poor endoscopic status (eg, Mayo or SES-CD) or adverse drug reactions. Unfortunately, repeat endoscopies were not completed by patients who underwent a second combination solely for endoscopic improvement.

### Laboratory Value Changes

Laboratory values measured pre- and postdual-targeted therapy included ESR, CRP, fecal calprotectin, hemoglobin, and albumin. There was a statistically significant improvement in average ESR (*P* = .006), average CRP (*P* < .001), and average albumin (*P* < .001). There were trends toward improvement in average fecal calprotectin (*P* = .153) and hemoglobin (*P* = .074), but these did not reach statistical significance. Laboratory outcomes are summarized in Table 6.

**Table 6.** Laboratory marker response to dual-targeted therapy.

Laboratory test	Precombination median (range, interquartile range)	Postcombination median (range, interquartile range)	*P*-value
ESR (mm/h)	19 (2-124, 28)	9 (0-116, 21)	.006^a^
CRP (mg/L)	8.0 (0.2-78.5, 15.6)	3.0 (0.2-68.2, 5.4)	<.001^a^
Fecal calprotectin (mcg/g)	378 (18-8000, 859)	192 (5-2430, 392)	.153
Hemoglobin (g/dL)	12.8 (7.8-16.1, 2.2)	13.2 (8.4-17.6, 1.8)	.074
Albumin (g/dL)	4.0 (2.2-4.9, 0.6)	4.2 (3.4-5.2, 0.6)	<.001^a^

^a^Denotes statistical significance.

### Complications, Hospitalizations, and Surgeries

Of the 97 dual-targeted therapy cases, there were 37 therapy-related adverse events. The most common complications were upper respiratory tract infections (*n* = 8, 21.6% of complications). Of note was that 2 cases of *Clostridium difficile* and 1 case of cytomegalovirus colitis were diagnosed while on dual-targeted therapy. The other most common type of complication was dermatologic, including 4 cases of dermatitis, 3 cases of acne, and 1 case of melanoma diagnosed while on dual-targeted therapy. There was 1 case of nonfatal anaphylaxis while on dual-targeted therapy (following a vedolizumab treatment) requiring a single epinephrine injection. There were no deaths related to dual-targeted therapy. A detailed breakdown of therapy complications is depicted in Table 7. Reasons for therapy discontinuation included lack of improvement (*n* = 22), insurance issues (*n* = 6), adverse reaction (*n* = 5), surgical intervention (*n* = 3), patient preference (*n* = 1), or for an unknown reason (*n* = 7).

**Table 7.** Complications and the associated combination therapy.

Type	Related complication	Frequency	Associated combinations (#)
Allergic	Anaphylaxis	1	vedolizumab + tofacitinib
Dermatologic	Dermatitis	4	ustekinumab + infliximab (1), ustekinuab + upadacitinib (1), vedolizumab + tofacitinib (2)
	Acne	3	ustekinumab + upadacitinib (3)
	Melanoma	1	ustekinumab + upadacitinib
Gastrointestinal	Pancreatitis	1	vedolizumab + tofacitinib
	Hepatic steatosis	1	ustekinumab + tofacitinib
	Elevated liver enzymes	1	ustekinumab + tofacitinib^a^
Hematologic	Leukopenia	2	ustekinumab + upadacitinib (1), vedolizumab + tofacitinib (1)
	Easy bruising	1	
Infectious	Oral candidiasis	1	vedolizumab + tofacitinib^b^
	Vulvovaginal candidiasis	1	vedolizumab + tofacitinib^b^
	Peri-umbilical abscess	1	vedolizumab + certolizumab
	Upper respiratory tract infection	8	ustekinumab + tofacitinib (3), ustekinumab + upadacitinib (2), vedolizumab + tofacitinib (1) vedolizumab + certolizumab (1), vedolizumab + upadacitinib (1)
	Otitis media	1	vedolizumab + tofacitinib
	Clostridium difficile colitis	2	vedolizumab + tofacitinib
	Salmonella gastroenteritis	1	ustekinumab + tofacitinib
	Herpes zoster	1	ustekinumab + upadacitinib
	Oral herpes simplex	1	vedolizumab + tofacitinib
	Cytomegalovirus colitis	1	ustekinumab + upadacitinib
Musculoskeletal	Arthralgia	1	vedolizumab + tofacitinib
Neurologic	Neuropathy	1	vedolizumab + tofacitinib
Psychiatric	Anxiety	1	vedolizumab + tofacitinib
Genitourinary	Erectile dysfunction	1	ustekinumab + upadacitinib

^a^Liver enzyme elevation resulted in discontinuation of the combination therapy.

^b^Candidal infections were confounded by the patient having finished a course of oral steroids.

There were 20 recorded IBD-related hospitalizations (eg, severe flares, bleeding, perforation, etc.) while patients were on dual-targeted therapy. Following the initiation of dual-targeted therapy, 15 surgical cases were performed. The most common surgeries were total colectomy (*n* = 5) or ileocolectomy (*n* = 3). The median time to surgery was 4.5 months (ranging from 0.5 to 36 months).

## Discussion

In this study, we report on the efficacy and safety of dual-targeted therapy with biological and small molecule therapy patients with IBD. The baseline characteristics of the patients who received dual-targeted therapy affirm that these patients are complex and with severe disease. Before dual-targeted therapy, most of the patients had at least one IBD-related hospitalization (79.7%), and a third had at least one IBD-related surgery (37.6%). Twenty patients had IBD-related hospitalizations on dual-targeted therapy. Fifteen patients required surgery within an average of 4.5 months after starting therapy. Furthermore, on average, nearly 3 biologics had been used for each patient before initiation of dual-targeted therapy (see Table 3).

Pritivera et al. proposed 2 reasons for initiating dual-targeted therapy. The first was for those with poorly controlled luminal disease. The idea was that adding a second agent could be used for those with inadequate response to a first agent or used both as induction therapy for those who had been exposed to multiple agents.^16,19^ The second reason is for those with uncontrolled extraintestinal manifestations or luminal disease on a single agent.^16,19^ These reasons guided our practice, and the 2 most common reasons for initiating dual-targeted therapy were to improve endoscopic disease and clinical gastrointestinal symptoms, which are the factors that recent guidelines use to define clinical remission.^20^ Overall, we had endoscopic improvement in 69.0% of matched cases and clinical improvement in 73.2% of matched cases (ie, out of the cases that had available data, 42 for endoscopic response and 82 for clinical response). This is further broken down into clinical improvement in 62.5% of dual biologic cases and 74.3% of biologic plus JAK inhibitor cases. Laboratory numbers associated with improvement in IBD generally trended towards improvement, with ESR, CRP, and albumin reaching statistical significance. Obesity was associated with worse outcomes, consistent with current literature. Obesity has been seen to impact clinical course with a lower prevalence of clinical remission, healthcare utilization, and worse surgical outcomes.^21–23^

Ahmed et al.’s meta-analysis from 2022 looked at 279 patients on dual biologic or small-molecule therapy and noted endoscopic remission rates of 34% and clinical remission rates of 59%.^9^ A meta-analysis from 2022 noted about 266 patients on dual-targeted therapy with biologics and small molecules.^10^ They found a clinical response rate of about 77.9% for those on vedolizumab/tofacitinib, 59.9% in those with vedolizumab and tofacitinib, and 83.9% for those on vedolizumab and ustekinumab.^10^

In terms of recent RCTs, these also support improved clinical efficacy with dual-targeted therapy in a controlled setting. It is important to note that the combinations have not been readily used in real-world data, and many of the patients are not the refractory patients for which dual-targeted therapy is being used. The VEGA trial used guselkumab and golimumab naive to an anti-TNF and refractory or intolerant to conventional therapy.^18^ While golimumab is approved for the treatment of IBD, it is rarely used and was not used within our cohort, and guselkumab has not been approved by the FDA for the treatment of IBD. However, this study also showed increased clinical efficacy with dual-targeted therapy (83%) compared to golimumab (61%) and guselkumab (75%) alone.^18^ The EXPLORER trial looked at the use of vedolizumab, adalimumab, and methotrexate therapy in biologic-naive patients and found no increased safety signals in triple therapy and endoscopic remission in 54.4%.^17^

The differences in the literature and our numbers may lie in the number of dual-targeted with dual biologic versus the biologic/small molecule combinations. Most of our patients were on combinations of biologics with small molecules rather than being on dual biologics. Most of the 97 cases were treated with a combination of a biologic and a JAK inhibitor (90.7%). The choice of biologic was usually either ustekinumab or vedolizumab given the gut-specific mechanism of action for those with uncontrolled luminal disease and, in theory, lower immunosuppressive capacity compared to anti-TNF therapy.^14,24,25^ Another reason ustekinumab and vedolizumab were the most common biologics used in our combination therapy was the fact that many patients had already failed anti-TNF therapy; 93.6% of patients were on TNF-alpha inhibitor therapy before dual-targeted therapy. There were only 2 patients who underwent dual-targeted therapy with an anti-TNF.

While there is data on small molecules and biological therapy, most of it relates to the use of tofacitinib. There is only one case series where 10 patients were treated with the use of upadacitinib and ustekinumab with 80% efficacy.^26^ Our data shows efficacy with the use of tofacitinib and upadacitinib in various combinations with biologics (Table 2). We also had combinations of therapy with risankizumab, and there is no other literature present at this time showing its efficacy in dual-targeted therapy.

Regarding safety, the main potentially related adverse events we noted were upper respiratory tract infections and dermatologic manifestations (each accounting for 8.2% of cases and 21.6% of complications). The upper respiratory infections and dermatological manifestations are lined up with known side effects of biological therapies. There was one case of melanoma that was diagnosed on dual-targeted therapy, and the only other serious side effect was nonfatal anaphylaxis on vedolizumab therapy. Side effects did lead to discontinuing dual-targeted therapy for 5 patients in our cohort. Reassuringly, there were no thromboembolic events and only one case of herpes zoster, both of which have been associated with the JAK2 inhibitors.^27^ Alayo et al. also did not report herpes zoster or deep vein thromboses in this study or in their study of dual-targeted therapy with tofacitinib and a biologic.^28^ The real-world tofacitinib monotherapy study in patients with UC reported an adverse event rate of 15.7%.^29^ Regarding dual-targeted combination therapy, the EXPLORER trial did not notice any increased safety signals, and the VEGA trial also reported comparable adverse event rates.^17,18^ The average time patients were on dual-targeted therapy was 39.1 weeks, and thus, it is possible that these side effects were not seen given the short time interval.

The reasons for discontinuing dual-targeted therapy varied; 22.7% of dual-targeted therapy discontinuation was attributed to treatment failure. From these cases, only 6 patients went on to another dual-targeted therapy, and 5/6 of these patients had improvement. Insurance barriers accounted for 6/97 (6.2%) cases of combination discontinuation. Cost remains a significant consideration. However, achieving disease remission would decrease costs in the long run.^30^

As with retrospective studies, there are limitations to this study. There was a lack of patient follow-up as 53.6% of dual-targeted therapy cases had endoscopies shown as ordered but never completed in the authors’ electronic medical record system. Furthermore, there was some variability in terms of time to endoscopy. Similar issues arose with imaging and laboratory results postcombination therapy. There were also limitations in terms of objectively defining clinical responses. Clinical improvement was graded based on a 2-author review of patient records and their response to changes in bowel movement frequency, hematochezia, and pain after dual-targeted therapy initiation with regards to their symptoms, rather than using a validated tool such as the U-IBDQ. Clinical improvement for extraintestinal manifestations was also assessed based on reported patient outcomes. Finally, most of our dual-targeted therapy cases involved a biologic plus a JAK inhibitor (88 cases, 90.7%), while only 9 (9.3%) cases included combination biologic therapy, making statistical analysis/comparison between these groups difficult. Our sample sizes were too small to determine which combinations were more efficacious. Still, given the heterogeneity of these complex patients, it is unclear how much more clarity this would provide.

Our paper adds information to this new treatment paradigm. While there are some small studies looking at dual-targeted therapy with combinations of biological therapy with small molecules, most of the literature focuses on combinations of 2 biologics. Our study involved a robust sample of varying combinations of mostly biologics with small molecules and investigated the safety, efficacy, and reasons for discontinuation. Our hope is that this will allow for more use of these combinations and trigger further research into this management paradigm.

In summary, our single-center experience does not show severe adverse effects and shows reasonable clinical efficacy and safety in treating patients with dual-targeted therapy for severe and refractory IBD.

## Data Availability

Data are not publicly available due to patient privacy concerns.
